# Predictive Effect of Positive Youth Development Attributes on Delinquency Among Adolescents in Mainland China

**DOI:** 10.3389/fpsyg.2020.615900

**Published:** 2020-12-14

**Authors:** Xiaoqin Zhu, Daniel T. L. Shek

**Affiliations:** Department of Applied Social Sciences, The Hong Kong Polytechnic University, Kowloon, Hong Kong

**Keywords:** longitudinal study, delinquency, life satisfaction, mediation, high school student

## Abstract

The general proposition of the positive youth development (PYD) approach is that developmental assets such as psychosocial competence can promote healthy adolescent development and reduce problem behavior. Despite that many Western studies have shown that PYD attributes are negatively related to adolescent delinquency, not all empirical findings support the negative associations. Although different dimensions of PYD attributes may bear differential relationships with delinquency, this possibility has not been properly examined so far. In addition, related studies in mainland China do not exist. Finally, the possible mediating role of life satisfaction in linking PYD attributes to delinquency has rarely been studied. To address the research gaps and understand how PYD attributes are associated with adolescent delinquency and the underlying mediating effect of life satisfaction, matched longitudinal data were collected from 2,648 mainland Chinese secondary school students (1,109 girls, Mean age = 13.12 ± 0.81 years at Wave 1) at two waves which were separated by one year. On each occasion, participants completed a questionnaire containing validated measures of PYD attributes, life satisfaction, and delinquency. Congruent with the general theoretical prediction of the PYD approach, different PYD attributes were inversely related to concurrent and future adolescent delinquency in separate regression analyses. In addition, the negative predictions were mediated by life satisfaction. When all PYD attributes were included in a single path analysis model, three findings were observed. First, two PYD dimensions, including self-identity and general PYD attributes, showed robust negative predictions on delinquency via life satisfaction. Second, prosocial attributes displayed a weak and unstable negative predictive effect. Third, cognitive-behavioral competence showed an unexpected positive predictive effect on delinquency directly or via its negative effects on life satisfaction. The present findings add value to the existing literature by revealing the predictive role of PYD attributes on life satisfaction and delinquency among mainland Chinese adolescents. The findings also reinforce the importance of investigating individual dimensions of PYD attributes simultaneously in the research field. The present study suggests that it is promising to cultivate PYD attributes as a strategy to reduce delinquency among adolescents in mainland China.

## Introduction

Adolescence is a transition period when adolescents experience physical and psychosocial changes, explore their adult identities, and learn to live independently. If adolescents are not capable of dealing with developmental challenges, problematic behaviors such as delinquency are likely to emerge. Plentiful evidence shows that higher prevalence rates of delinquency are growing global concern, particularly among early adolescents in both Western and Chinese contexts (Felson and Kreager, 2015; Shek and Lin, 2016). Delinquency’s co-occurrence with other developmental issues, such as depression and substance consumption, also severely hinders interpersonal development, academic achievement, well-being, and even society’s sustainability (Chen et al., 2012; McDonough-Caplan et al., 2018). In fact, early and persistent delinquent behaviors have been regarded as strong predictors of later violence, unemployment, and substance abuse (Bradshaw et al., 2010; Brook et al., 2013). For example, adolescents who were involved in battling and inferior theft reported maladaptive problems such as depression, withdrawal from high school in late adolescence, and substance abuse problems (Cook et al., 2015). In view of these long-term disruptive outcomes, adolescent delinquency has brought heavy stresses and costs to families and society (Regoli et al., 2016). Thus, identifying factors that protect adolescents from delinquency becomes an important task of youth studies.

Although research and intervention programs have long adopted a problem-centered approach which focuses on developmental deficits and “treating the problems,” most youths experience adolescence with promising and positive trajectories, despite the thorough physiological, behavioral, and psychosocial changes across the period (Lerner, 2009). Some scholars believe that adolescents are not “troubles” but valuable resources with capability, potential, and strength that can be nurtured and utilized for their holistic development and positive functioning (Damon and Gregory, 2003). With such an emphasis on adolescent strengths, the positive youth development (PYD) approach has been used to understand adolescent development and the importance of nurturing their positive attributes (Tolan et al., 2016). In contrast to the deficit perspective highlighting developmental risks, the PYD perspective argues that various youth problems, including delinquency, can be mitigated or avoided through the cultivation of PYD attributes (Shek et al., 2019a).

Theoretically, PYD attributes are a set of developmental assets related to one’s inner world and positive experience derived from the external world, both of which can be utilized to help adolescents effectively cope with developmental challenges and buffer life stress, thus reducing problem behavior and making adolescents thrive in adversity (Shek et al., 2019a). Scholars have conceptualized PYD attributes from different approaches. For instance, the developmental assets framework proposed by Benson et al. (2011) holds that there are 20 internal assets (positive individual strengths such as positive values and social competencies) and 20 external assets (supportive environment and constructive interactions with the external world such as empowering and expectations) that are critical for youth growth and thriving. Similarly, Lerner et al. (2011) emphasized the importance of “Five Cs,” including “connection,” “confidence,” “competence,” “character,” and “caring” in healthy youth development. The authors further pointed out that the development of these five Cs in adolescents will eventually shape the sixth C, “contribution.” In addition, Catalano et al. (2004) summarized 15 essential PYD constructs that had been widely incorporated in effective youth prevention programs. These PYD constructs cover a wide range of positive internal assets, such as cognitive, emotional, and social skills, prosocial values, optimism about the future, positive self-perception, optimism, and spirituality, as well as positive external assets, such as bonding with parents, teachers, and friends, and the supportive environment for doing prosocial behavior.

Consistent with the general theoretical expectation that PYD leads to good developmental outcomes, the above mentioned PYD attributes defined in different frameworks have been empirically found to be protective factors against adolescent delinquency. Specifically, both the global measures of PYD or individual PYD attributes negatively predict delinquency among children and adolescents. For example, Geldhof et al. (2014) reported that both the individual “Five Cs” (i.e., “connection,” “confidence,” “competence,” “character,” and “caring”) and the integrated higher-order of the “Five Cs” negatively correlate with the composite problem behavior including delinquency and substance use among American adolescents. Likewise, Sun (2016) found that PYD attributes indexed by Catalano’s 15 PYD constructs also significantly and negatively predict misconduct in Hong Kong Chinese adolescents. Different PYD attributes have also been identified to be negatively associated with delinquency among mainland Chinese adolescents (Huang et al., 2019; Chai et al., 2020). Evaluation findings from youth programs adopting PYD approaches in the West and Chinese contexts also demonstrated that the cultivation of multiple PYD attributes among adolescents successfully lessens the likelihood of their participation in problem behavior, including delinquency (Domitrovich et al., 2017; Ma et al., 2019; Zhu and Shek, 2020a).

Despite the accumulating evidence that indicates the inverse relationship between PYD attributes and delinquency, several research gaps are present. First, not all empirical findings showed significant negative associations between PYD attributes and delinquency. For instance, Phelps et al. (2007) reported that youth with the highest trajectory of PYD attributes tended to display the up-and-then-down trajectory in risk behavior. Lewin-Bizan et al. (2010b) did not find an inverse relationship between PYD and problematic behaviors (including delinquency and substance use) among most youth. Specifically, some adolescents with a decreasing PYD trend were more likely to exhibit a low trajectory rather than an increase in problematic behaviors. Shek and Lin (2016) also demonstrated a positive relationship between PYD and an increased rate of delinquency among Chinese adolescents. Noteworthy, PYD attributes in all the above-mentioned studies were indexed by a global measure, thus ignoring the nuanced relationship between individual dimensions of PYD and delinquency as well as the possibility that the individual dimensions may bear differential relationships with delinquency (Arbeit et al., 2014; Geldhof et al., 2014). Therefore, it is necessary to clarify the relationships between PYD and delinquency by using not only a global measure of PYD but also individual dimensions underlying the PYD construct.

Second, the potential mediating mechanisms underlying the link between PYD attributes and adolescent delinquency have been under-researched. In particular, life satisfaction, which refers to one’s cognitive evaluation of his or her overall life quality (Diener et al., 1985), may serve as a mediator in linking PYD attributes and delinquent behavior among adolescents. Theoretically, the development of positive psychosocial competence enables adolescents to well adapt to biological, psychological, and social changes occurring in adolescence (Larson, 2011). With the healthy adjustment, adolescents are more likely to live a healthy lifestyle, maintain health and fitness, gain support and build positive connections, all of which make adolescents positively appraise their life (Shek et al., 2019a; Ma, 2020). A higher level of life satisfaction, in turn, cultivates a positive appraisal style, which renders adolescents to copy stressful life events and environmental challenges more effectively (Shek and Chai, 2020) and less likely to externalize life distress to delinquent behavior (Park, 2004).

The above theoretical notions have been supported by rich empirical findings showing positive predictions of PYD attributes on adolescent life satisfaction (Zhang, 2016; Lázaro-Visa et al., 2019; Ma, 2020) and negative predictions of life satisfaction on adolescent delinquency (Jung and Choi, 2017; Hanniball et al., 2018; Lee et al., 2020). A handful of pioneering studies have empirically tested the mediational role of life satisfaction in linking PYD attributes to behavioral problems (Sun and Shek, 2010, 2012). Nevertheless, these studies only used global indicators of PYD without considering individual dimensions. As a result, there is a need to further clarify the proposed mediating effects of life satisfaction on the associations between individual PYD dimensions and delinquency among adolescents.

Third, while there are many Western studies on PYD attributes and adolescent risk behavior, very few systematic studies have been carried out to understand how PYD attributes are associated with adolescent delinquency in mainland China (Wiium and Dimitrova, 2019). Primarily, the function of PYD attributes may not be the same in different cultural contexts (Wiium and Dimitrova, 2019), especially concerning the differences in the emphasis on positive youth attributes between Western and Chinese contexts. For example, some PYD constructs conceived in the West are closely related to individualistic “Me” and autonomous self, such as self-identity, self-efficacy, and self-determination. However, these qualities are not strongly emphasized in traditional Chinese thoughts, which place more emphasis on interdependent “We” and relational harmony (Yang and Zhou, 2017). Empirically, studies reported unique definitional and structural features of PYD attributes among mainland Chinese adolescents, and such features are different from that have been identified in models based on Western adolescents (Chen et al., 2018; Chai et al., 2020). For example, the individual-individual connection (i.e., peer connection) among mainland Chinese adolescents is not as salient as that in their Western counterparts, which may be attributable to the collectivistic orientation in China where the individual-level connection is likely to be embedded in the individual-group connections in family, school, and society (Chai et al., 2020).

Nonetheless, internal and external assets are also highlighted in Chinese traditions. In Confucian thoughts, the development of virtues is an important factor shaping the social behavior of children. By cultivating virtues and good character in children (i.e., internal assets), children would thrive (Shek et al., 2013). At the same time, positive environmental influence (i.e., external asset) was emphasized by Mencius, as exemplified by the “three moves” of the mother of Mencius (Carey and Vitz, 2020). Furthermore, the rapid social and economic development in mainland China makes Chinese people more and more “Westernized.” For instance, individualistic values have been increasingly adopted in Chinese society (Steele and Lynch, 2013). In particular, Chinese parents have adapted to the globalization and westernization by confirming less to traditionally prescribed parental roles and beliefs as well as stressing more on the development of children’s independence and self-expression (Chang et al., 2011; Inglehart et al., 2020). It has also been found that the younger generation in mainland China are more self-centered and individualistic but less identified with the traditional collective ideology than the old generations (Sun and Wang, 2010; Zhou et al., 2018). In view of these contextual features in mainland China, it will be theoretically illuminating to examine the inter-relationships among PYD attributes, life satisfaction, and delinquency among mainland Chinese adolescents. In addition, among the world adolescent population aged between 10 and 19, roughly 13.5% are Chinese adolescents in mainland China (UNICEF, 2019), which makes Chinese data essential to establishing the universality of the related findings in the field of PYD studies.

To address these research gaps, this study attempted to provide answers to the following three research questions using two waves of data collected from Chinese adolescents in mainland China.

Research Question 1: What are the predictions of PYD attributes on adolescent delinquency? Based on the theoretical frameworks of PYD approaches, we expected negative concurrent and longitudinal predictions of PYD attributes on adolescent delinquency (Hypothesis 1).

Research Question 2: Does life satisfaction function as a mediator underlying the linkage between PYD attributes and adolescent delinquency? Based on the preceding literature review, we hypothesized that life satisfaction serves as the mediator for the predictive effects of PYD attributes on delinquency. We expected that PYD attributes positively predict life satisfaction (Hypothesis 2), which in turn negatively predicts adolescent delinquency (Hypothesis 3).

Research Question 3: Do the hypothesized relationships differ for the global measure and the individual dimensions of PYD attributes? Despite the general theoretical prediction of a negative relationship between PYD attributes and delinquency, some empirical findings suggest that individual PYD dimensions may bear differential associations with delinquency (Arbeit et al., 2014; Geldhof et al., 2014), leading to different predictions between the global PYD measure and individual PYD dimensions. Therefore, in addition to investigating the influence of individual dimensions and the global PYD measure on delinquency separately, we also included the different PYD dimensions in a single model to reveal their relative influence.

**FIGURE 1:**
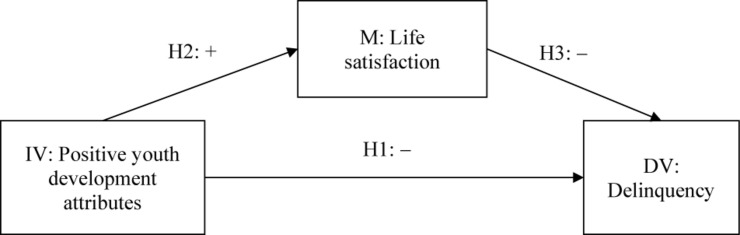
Hypothesized relationships among positive youth development attributes, life satisfaction, and delinquency. IV, independent variable; M, mediator; DV, dependent variable.

Based on the above hypotheses, we proposed a mediating effect model among PYD attributes, life satisfaction, and adolescent delinquency as the conceptual framework of the present study (see Figure 1). In the mediation model, both the global measure and individual dimensions of PYD attributes were examined. In the previous studies, age, gender, and family intactness were found to be associated with adolescent life satisfaction and problem behavior (Proctor et al., 2009; Chen, 2010; Sogar, 2017). Hence, these demographic variables were included as control variables in this study.

## Materials and Methods

### Participants and Procedures

This study was a 2-year survey involving four junior secondary schools in four different cities (Suzhou, Jiujiang, Zhaoqing, and Shanwei) in mainland China. The first occasion of data collection was conducted at the beginning of the 1^st^ semester in the 2016/17 school year. All the Grade 7 and Grade 8 students in these schools were invited to respond to questionnaires measuring adolescent PYD attributes, problem behavior (e.g., delinquency), and well-being. One year later, these students responded to the questionnaires once again.

At Wave 1, a total of 3,010 (*n* = 1,362 at Grade 7; *n* = 1,648 at Grade 8) students completed the survey, among whom, 2,648 students (*n* = 1,305 at Grade 7; *n* = 1,343 at Grade 8) also completed the survey at Wave 2, resulting in an overall attrition rate of 12.03%. The matched sample (*N* = 2,648, Mean age = 13.12 ± 0.81 years at Wave 1), who completed the questionnaires at both waves, formed the working sample of this study. Among these students, 1,109 (41.88%) were girls, 1,513 (57.14%) were boys, and 26 students (0.98%) did not report their gender information. A total of 2,225 (84.03%) students were from intact families, and the other 401 (15.14%) students reported that they were living in non-intact families.

Ethical approval was obtained from the “Human Subjects Ethics Subcommittee” at the authors’ university. Before the commencement of the study, the participating schools and parents of the students gave their written consent for the students’ participation after we explained the study purposes and key principles we would follow in collecting, using, and disseminating data. These principles included voluntary participation, do-no-harm, anonymity, confidentiality, and free withdrawal. Prior to each occasion, all student respondents signed the written consent after they were well informed of the study objectives and those aforementioned vital principles.

### Measures

The survey contained several validated measures related to the psychological adjustment of adolescents. The foci of the present study were the associations among PYD attributes, life satisfaction, and delinquency. The measurement tools of these constructs are outlined below.

#### PYD Attributes

The 80-item “The Chinese Positive Youth Development Scale” (CPYDS) was adopted in this study. This scale was developed and validated in a local context (Shek and Ma, 2010), and it has demonstrated good psychometric properties in assessing PYD attributes among Chinese adolescents in prior research (Sun and Shek, 2012; Sun, 2016; Zhu and Shek, 2020a). There were 15 subscales in the CPYDS assessing the key 15 PYD attributes (e.g., emotional competence, resilience, and prosocial involvement) included in Catalano et al.’s (2004) PYD framework. These 15 primary PYD attributes were further grouped into four individual PYD dimensions including (1) Cognitive-behavioral competence (CBC) under which there were three primary qualities, i.e., “cognitive competence,” “self-determination,” and “behavioral competence”; (2) Prosocial attribute (PA) which consisted of two primary qualities, i.e., “prosocial involvement” and “prosocial norms”; (3) Positive identity (PI) that included two primary qualities (i.e., “clear and healthy identity” and “beliefs in the future”); and (4) General PYD attribute (GPYD) under which there were eight primary qualities, including “bonding,” “social competence,” “emotional competence,” “moral competence,” “resilience,” “self-efficacy,” “spirituality,” and “recognition for positive behavior.” All items were rated from 1 (“strongly disagree”) to 6 (“strongly agree”). Confirmatory factor analyses (CFA) showed that the hierarchical factor structure with four higher-order factors indicated by 15 primary factors showed acceptable model fit at Wave 1 (χ*^2^* = 9834.88, *df* = 3048, CFI = 0.90, NNFI = 0.89, RMSEA = 0.03, average primary factor loading = 0.65, average higher-order factor loading = 0.86) and Wave 2 (χ*^2^* = 11044.81, *df* = 3048, CFI = 0.90, NNFI = 0.90, RMSEA = 0.03, average primary factor loading = 0.72, average higher-order factor loading = 0.87). Composite scores were computed for the four individual PYD dimensions. Besides, a total PYD (TPYD) score as a global measure of PYD was also calculated by averaging scores across all items. The related Cronbach’s alpha values can be seen in Table 1.

**TABLE 1:** Descriptive statistics and reliability measures for positive youth development attributes, life satisfaction, and delinquency.

**Measures**	**Wave 1**				**Wave 2**			
	**Mean**	***SD***	**α**	**Mean inter-item correlation**	**Mean**	***SD***	**α**	**Mean inter-item correlation**
Positive youth development attributes								
CBC	4.82	0.74	0.91	0.38	4.95	0.78	0.94	0.49
PA	4.87	0.84	0.86	0.40	5.01	0.85	0.90	0.48
PI	4.73	1.00	0.88	0.44	4.87	1.02	0.90	0.49
GPYD	4.81	0.71	0.95	0.30	4.92	0.76	0.96	0.37
TPYD	4.78	0.69	0.97	0.31	4.90	0.73	0.98	0.38
Life satisfaction	4.06	1.12	0.81	0.48	4.06	1.13	0.84	0.53
Delinquency	0.45	0.54	0.74	0.27	0.42	0.57	0.78	0.34

*CBC, cognitive-behavioral competence; PA, prosocial attribute; PI, positive identity; GPYD, general positive youth development attribute; TPYD, total positive youth development attribute*.

#### Life Satisfaction (LS)

Life satisfaction was measured by the “Satisfaction with Life Scale” (Diener et al., 1985), which has been locally validated by Shek (1992) for assessing Chinese people’s evaluation of their overall LS (Zhu and Shek, 2020b). The scale included five items. Using a rating scale with six points (“1 = strongly disagree”; “6 = strongly agree”), the respondents indicated their cognitive evaluations of their overall life quality (e.g., “The conditions of my life are excellent” and “I am satisfied with my life”). In this study, CFA yielded good model fit for the single-factor structure of LS across waves (Wave 1: χ*^2^* = 58.89, *df* = 4, CFI = 0.99, NNFI = 0.97, RMSEA = 0.07, average factor loading = 0.67; Wave 2: χ*^2^* = 72.72, *df* = 4, CFI = 0.99, NNFI = 0.97, RMSEA = 0.08, average factor loading = 0.71). The Cronbach’s α estimates of the scale were above 0.80 across the two waves (see Table 1).

#### Delinquency

A 12-item scale was used to assess how often (“0 = never”; “6 = more than 10 times”) the participants engaged in the listed twelve delinquent behaviors, such as “stealing,” “cheating,” “running away from home,” “staying outside the home overnight without parental consent,” and “trespassing,” during the last 12 months. Among these behaviors, some (e.g., “stealing” and “damaging others’ properties”) can be considered illegal, while other behaviors such as “running away from home” and “having sexual intercourse with others” do not violate the law but are perceived to be risky for adolescents in the Chinese context. The one-factor structure of this scale possessed adequate reliability and validity in measuring Chinese adolescents’ delinquency in previous research (Shek and Zhu, 2019). In the present study, CFA showed adequate model fit for the one-factor model of delinquency at both waves (Wave 1: χ*^2^* = 778.74, *df* = 49, CFI = 0.90, NNFI = 0.90, RMSEA = 0.07, average factor loading = 0.51; Wave 2: χ*^2^* = 838.10, *df* = 49, CFI = 0.93, NNFI = 0.90, RMSEA = 0.08, average factor loading = 0.58). The Cronbach’s α value was 0.74 and 0.78 at the two assessment occasions, respectively (see Table 1).

#### Covariates

Consistent with previous studies, gender, age, and family intactness were measured as covariates. Intact families were defined as families in which parents were in their first marriage, while non-intact families were characterized by parental separation, divorce, or re-marriage.

### Data Analysis

First, correlational analyses were conducted to check the cross-sectional and longitudinal correlations among key variables. Second, to investigate the hypothesized relationships among PYD attributes, life satisfaction, and delinquency, we used the PROCESS macro in SPSS 26.0 (Preacher and Hayes, 2008) to test the mediation model displayed in Figure 1. Five separate mediating effect analyses were carried out based on the four higher-order PYD attributes and the total PYD score, respectively. Third, to further investigate the proposed mediation model in a more holistic manner, we also performed path analysis using structural equation modeling (SEM), including four higher-order PYD attributes simultaneously in one single model. The path analysis was performed using AMOS 26.0. Model fit indices, including “Comparative Fit Index” (CFI), “Non-Normed Fit Index” (NNFI), and “Root Mean Square Error of Approximation” (RMSEA), were utilized to assess model fit. CFI > 0.90, NNFI > 0.90, and RMSEA < 0.08 indicate an adequate model fit (Kline, 2015). Following suggestions given by Preacher and Hayes (2008), we performed bootstrapping with 5,000 resamples in both PROCESS and path analysis and calculated bias-corrected (BC) 95% confidence intervals (CIs).

## Results

### Attrition Analyses

Results revealed that the matched sample (*N* = 2,648) and those 362 dropouts did not differ in their demographic attributes, including age, gender, and family intactness. Regarding baseline PYD attributes, results showed that dropouts at Wave 2 reported higher scores on GPYD and the total PYD score than did non-dropouts at the same grade level (mean differences = 0.15–0.23, *t* = 2.28–3.80, *p*s < 0.001, Cohen’s *d* = 0.22–0.33). While the two groups at Grade 7 did not significantly differ from each other on baseline LS, Grade 8 dropouts (*M* = 4.30, *SD* = 1.10) showed higher baseline life satisfaction than did non-drops (*M* = 3.98, *SD* = 1.07; *t* = 4.59, *p* < 0.001, Cohen’s *d* = 0.29). For baseline delinquency, the two groups did not have a significant difference. Because the differences between the two groups of students were not great, sample attrition was not a major concern.

### Correlations Among Variables

Correlations among variables are shown in Table 2. After Bonferroni-correction (*p* = 0.05/17 = 0.003), all PYD attributes were inversely correlated with delinquency and positively correlated with life satisfaction, both cross-sectionally and longitudinally. Besides, life satisfaction and delinquency were negatively correlated with each other, both cross-sectional and over time. Overall speaking, these observations are consistent with our original expectations.

**TABLE 2:** Correlations among control variables, positive youth development attributes, life satisfaction, and delinquency at two waves.

**Measures**	**Correlations**															
	**1**	**2**	**3**	**4**	**5**	**6**	**7**	**8**	**9**	**10**	**11**	**12**	**13**	**14**	**15**	**16**
(1) Age	–			–												
(2) Gender^*a*^	−0.08***	–														
(3) Family intactness^*b*^	0.02	0.01	–													
(4) W1 CBC	−0.09***	–0.01	−0.05**	–												
(5) W1 PA	−0.09***	0.08***	−0.05*	0.66***	–											
(6) W1 PI	−0.11***	−0.10***	−0.07***	0.71***	0.58***	–										
(7) W1 GPYD	−0.12***	0.01	−0.07***	0.83***	0.70***	0.74***	–									
(8) W1 TPYD	−0.12***	–0.01	−0.07***	0.90***	0.79***	0.83***	0.97***	–								
(9) W1 LS	−0.07***	−0.07***	−0.07***	0.51***	0.47***	0.56***	0.60***	0.61***	–							
(10) W1 DE	0.11***	−0.14***	0.08***	−0.29***	−0.31***	−0.26***	−0.36***	−0.35***	−0.29***	–						
(11) W2 CBC	−0.10***	–0.02	–0.02	0.44***	0.35***	0.39***	0.43***	0.45***	0.31***	−0.21***	–					
(12) W2 PA	−0.09***	0.07***	–0.02	0.34***	0.41***	0.32***	0.40***	0.41***	0.27***	−0.26***	0.65***	–				
(13) W2 PI	−0.06**	−0.12***	−0.04*	0.38***	0.30***	0.48***	0.41***	0.44***	0.34***	−0.18***	0.71***	0.61***	–			
(14) W2 GPYD	−0.08***	–0.01	–0.03	0.43***	0.38***	0.42***	0.49***	0.50***	0.38***	−0.25***	0.84***	0.69***	0.75***	–		
(15) W2 TPYD	−0.09***	–0.02	–0.03	0.45***	0.40***	0.45***	0.50***	0.51***	0.38***	−0.26***	0.91***	0.78***	0.83***	0.97***	–	
(16) W2 LS	–0.03	−0.08***	−0.06**	0.21***	0.18***	0.25***	0.27***	0.27***	0.38***	−0.16***	0.44***	0.43***	0.56***	0.57***	0.57***	–
(17) W2 DE	0.07***	−0.12***	0.06**	−0.21***	−0.20***	−0.17***	−0.23***	−0.23***	−0.21***	0.43***	−0.25***	−0.22***	−0.21***	−0.29***	−0.29***	−0.22***

^a^1 = male, 2 = female; ^*b*^1 = intact, 2 = non-intact; W1, Wave 1; W2, Wave 2; CBC, cognitive-behavioral competence; PA, prosocial attribute; PI, positive identity; GPYD, general positive youth development attribute; TPYD, total positive youth development attribute; LS, life satisfaction; DE, delinquency. *p < 0.05, **p < 0.01, ***p < 0.001.

### Predictions of PYD Attributes and Mediating Effect of Life Satisfaction

Tables 3, 4 show the results of cross-sectional and longitudinal mediating effect analyses through the PROCESS, respectively. Several observations can be highlighted. First, each PYD attribute showed significant and negative concurrent and longitudinal predictive effects on adolescent delinquency (i.e., total effect), providing support for Hypothesis 1. Second, all PYD attributes showed significant and positive concurrent and longitudinal predictive effects on life satisfaction, giving support for Hypothesis 2. Third, life satisfaction significantly and negatively predicted adolescent delinquency, showing support for Hypothesis 3. Finally, the indirect effects of each PYD attribute on concurrent or future delinquency via life satisfaction were also significant, supporting the mediating effect model. Overall speaking, the findings suggest that adolescents’ PYD attributes enable them to feel more satisfied with life, which in turn leads to a lower level of delinquency.

**TABLE 3:** Cross-sectional mediating effect analyses of life satisfaction (the mediator) at Wave 1 on the effect of PYD measures on delinquency at Wave 1.

	**Independent variables (IV)**														
	**CBC**			**PA**			**PI**			**GPYD**			**TPYD**		
**Regression**															
**models summary**	***B***	***SE***	***t***	***B***	***SE***	***t***	***B***	***SE***	***t***	***B***	***SE***	***t***	***B***	***SE***	***t***
Total effect of IV on DV	–0.20	0.01	−15.52***	–0.19	0.01	−16.39***	–0.14	0.01	−13.76***	–0.26	0.01	−19.11***	–0.26	0.01	−19.06***
IV to Mediator	0.77	0.03	30.46***	0.64	0.02	27.45***	0.67	0.02	34.30***	0.96	0.02	38.70***	1.00	0.03	40.00***
Mediator to DV	–0.09	0.01	−9.12***	–0.09	0.01	−9.17***	–0.10	0.01	−9.61***	–0.06	0.01	−5.66***	–0.06	0.01	−5.45***
Direct effect of IV on DV	–0.13	0.02	−8.88***	–0.13	0.01	−10.33***	–0.08	0.01	−6.27***	–0.20	0.02	−11.92***	–0.20	0.02	−11.74***

**Mediating**	**Point**	**Bootstrapping**		**Point**	**Bootstrapping**		**Point**	**Bootstrapping**		**Point**	**Bootstrapping**		**Point**	**Bootstrapping**	
**effect**	**estimate**	**(BC 95% CI)**		**estimate**	**(BC 95% CI)**		**estimate**	**(BC 95% CI)**		**estimate**	**(BC 95% CI)**		**estimate**	**(BC 95% CI)**	
		**Lower**	**Upper**		**Lower**	**Upper**		**Lower**	**Upper**		**Lower**	**Upper**		**Lower**	**Upper**
	−0.07***	–0.09	–0.05	−0.06***	–0.07	–0.04	−0.07***	–0.09	–0.05	−0.06***	–0.09	–0.03	−0.06***	–0.10	–0.03

Results pattern of mediating effect analyses at Wave 2 was the same as that shown in the table; in all analyses, covariates were statistically controlled. CBC, cognitive-behavioral competence; PA, prosocial attribute; PI, positive identity; GPYD, general positive youth development attribute; TPYD, total positive youth development attribute; BC, bias corrected; CI, confidence interval. ***p < 0.001.

**TABLE 4:** Longitudinal mediating effect analyses of life satisfaction at Wave 2 (the mediator) for the effect of PYD measures at Wave 1 on delinquency at Wave 2.

	**Independent variables (IV) at Wave 1**														
	**CBC**			**PA**			**PI**			**GPYD**			**TPYD**		
**Regression**															
**models summary**	***B***	***SE***	***t***	***B***	***SE***	***t***	***B***	***SE***	***t***	***B***	***SE***	***t***	***B***	***SE***	***t***
Total effect of IV on DV	–0.15	0.01	−10.42***	–0.13	0.01	−9.99***	–0.10	0.01	−8.85***	–0.18	0.02	−12.03***	–0.19	0.02	−12.02***
IV to mediator	0.32	0.03	10.83***	0.24	0.03	9.09***	0.30	0.02	13.06***	0.44	0.03	14.46***	0.44	0.03	14.14***
Mediator to DV	–0.09	0.01	−9.71***	–0.10	0.01	−10.08***	–0.09	0.01	−9.70***	–0.08	0.01	−8.72***	–0.08	0.01	−8.78***
Direct effect of IV on DV	–0.12	0.01	−8.35***	–0.11	0.01	−8.26***	–0.07	0.01	−6.31***	–0.14	0.02	−9.36***	–0.15	0.02	−9.41***

**Mediating**	**Point**	**Bootstrapping**		**Point**	**Bootstrapping**		**Point**	**Bootstrapping**		**Point**	**Bootstrapping**		**Point**	**Bootstrapping**	
**effect**	**estimate**	**(BC 95% CI)**		**estimate**	**(BC 95% CI)**		**estimate**	**(BC 95% CI)**		**estimate**	**(BC 95% CI)**		**estimate**	**(BC 95% CI)**	
		**Lower**	**Upper**		**Lower**	**Upper**		**Lower**	**Upper**		**Lower**	**Upper**		**Lower**	**Upper**
	−0.03***	–0.04	–0.02	−0.02***	–0.03	–0.02	−0.03***	–0.04	–0.02	−0.04***	–0.05	–0.03	−0.04***	–0.05	–0.03

In all analyses, covariates were statistically controlled. CBC, cognitive-behavioral competence; PA, prosocial attribute; PI, positive identity; GPYD, general positive youth development attribute; TPYD, total positive youth development attribute; BC, bias corrected; CI, confidence interval. ***p < 0.001.

**FIGURE 2:**
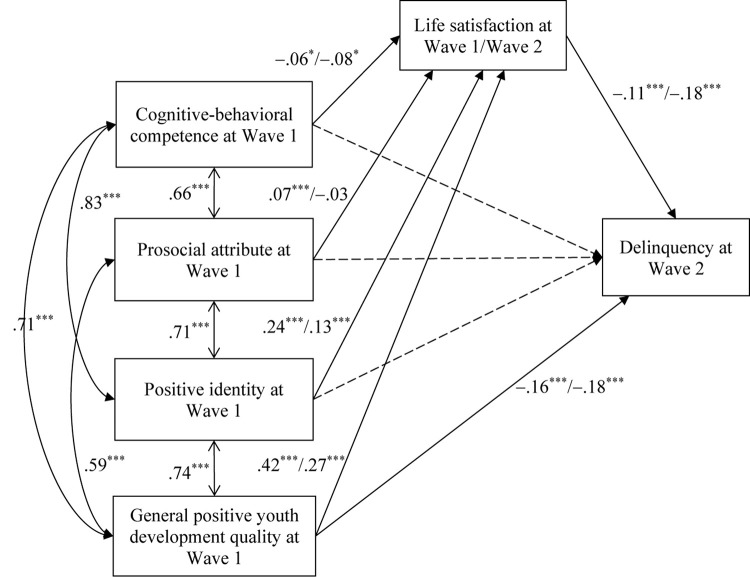
Standardized results of path analyses on the relationships among adolescent positive youth development attributes, life satisfaction, and delinquency. Age, gender, and family intactness were statistically controlled. Numbers before the slash are the results of analysis using life satisfaction at Wave 1 as the mediator and numbers after the slash are the results of analysis using life satisfaction at Wave 2 as the mediator. Solid paths indicate significant associations and dotted paths indicated insignificant associations. ^∗^*p* < 0.05, ^∗∗∗^*p* < 0.001.

Figure 2 outlines the significant standardized path coefficients of the mediation models involving the four higher-order PYD attributes simultaneously. The path coefficients and indirect effects of PYD attributes on delinquency are also presented in Table 5. We tested two models. In the first model, PYD attributes at Wave 1 were the independent variables (IVs), life satisfaction at Wave 1 was the mediator, and adolescent delinquency at Wave 2 was the dependent variable (DV). The second model used the same IVs and DV and used life satisfaction at Wave 2 as the mediator. The two models showed adequate model fit (the first model: χ*^2^* = 6.37, *df* = 3, χ*^2^/df* = 2.13, CFI = 0.99, NNFI = 0.99, RMSEA = 0.02; the second model: χ*^2^* = 9.48, *df* = 3, χ*^2^/df* = 3.16, CFI = 0.99, NNFI = 0.99, RMSEA = 0.03).

**TABLE 5:** Results of path analyses for the mediation model involving the four individual PYD attributes, life satisfaction, and delinquency.

**Paths**	***B***	***SE***	**β**
**W1 life satisfaction as the mediator**			
W1 CBC → W1 life satisfaction	–0.09	0.04	−0.06*
W1 PA → W1 life satisfaction	0.10	0.03	0.07***
W1 PI → W1 life satisfaction	0.29	0.03	0.25***
W1 GPYD → W1 life satisfaction	0.66	0.05	0.42***
W1 life satisfaction → W2 delinquency	–0.06	0.01	−0.11***
W1 GPYD → W2 delinquency	–0.13	0.02	−0.16***

		**Bootstrapping (BC 95% CI)**	
**Indirect effect on W2 delinquency through W1 life satisfaction**	**β**	**Lower**	**Higher**

W1 CBC	0.01	0.00	0.02
W1 PA	−0.01**	–0.02	–0.002
W1 PI	−0.03**	–0.40	–0.02
W1 GPYD	−0.05**	–0.70	–0.03

**Direct effect on W2 delinquency**			
W1 GPYD	−0.16**	–0.21	–0.11

***W2 Life satisfaction as the mediator***	***B***	***SE***	**β**

W1 CBC → W2 life satisfaction	–0.13	0.05	−0.08*
W1 PA → W2 life satisfaction	–0.05	0.04	–0.03
W1 PI → W2 life satisfaction	0.16	0.04	0.13***
W1 GPYD → W2 life satisfaction	0.44	0.06	0.28***
W2 life satisfaction → W2 delinquency	–0.90	0.01	−0.18***
W1 GPYD → W2 delinquency	–0.15	0.02	−0.18***

		**Bootstrapping (BC 95% CI)**	
**Indirect effect on W2 delinquency through W2 life satisfaction**	**β**	**Lower**	**Higher**

W1 CBC	0.02**	0.003	0.03
W1 PA	0.01	–0.003	0.02
W1 PI	−0.02**	–0.04	–0.01
W1 GPYD	−0.05**	–0.07	–0.03

**Direct effect on W2 delinquency**			

W1 GPYD	−0.18**	–0.22	–0.14

W1, Wave 1; W2, Wave 2; CBC, cognitive-behavioral competence; PA, prosocial attribute; PI, positive identity; GPYD, general positive youth development attribute; BC, bias corrected; CI, confidence interval. *p < 0.05, **p < 0.01, ***p < 0.001.

When using life satisfaction at Wave 1 as the mediator (i.e., the first model, see Table 5 and the results before the slash in Figure 2), three PYD attributes including prosocial attribute (β = 0.07, *p* < 0.05), positive identity (β = 0.24, *p* < 0.001) and general PYD attribute (β = 0.42, *p* < 0.001) were positive predictors of life satisfaction, which in turn negatively predicted adolescent delinquency (β = −0.11, *p* < 0.001). Thus, the negative indirect effects of these three PYD attributes on delinquency were significant (prosocial attribute: β = −0.01, *p* < 0.01, 95% CI = [−0.02, −0.002]; positive identity: β = −0.03, *p* < 0.01, 95% CI = [−0.04, −0.02]; general PYD quality: β = −0.05, *p* < 0.01, 95% CI = [−0.07, −0.03]). An unexpected finding is that cognitive-behavioral competence showed a negative predictive effect on life satisfaction (β = −0.06, *p* < 0.05). However, its indirect effect on delinquency was insignificant (β = 0.01, *p* > 0.05, 95% CI = [0.00, 0.02]). Besides, only general PYD attribute showed a significant direct effect on adolescent delinquency (β = −0.16, *p* < 0.01, 95% CI = [−0.21, −0.11]).

Similar observations were found when using life satisfaction at Wave 2 as the mediator (see Table 5 and the results after the slash in Figure 2). The only exception is that the prosocial attribute at Wave 1 did not show a significant longitudinal effect on life satisfaction at Wave 2 (β = −0.03, *p* > 0.05).

## Discussion

Based on a 2-year longitudinal design, this study investigated how PYD attributes were associated with adolescent delinquency and how life satisfaction mediated the effect among mainland Chinese adolescents. Consistent with the original predictions, single PYD attributes negatively predicted adolescent delinquency concurrently and longitudinally. As expected, life satisfaction also showed a significant mediating effect by being positively predicted by PYD attributes and negatively predicting delinquency. However, when all PYD measures were included in a single model, some “odd” observations for the role of cognitive-behavioral competence were found. The present study adds theoretical and practical value to the existing literature by deepening our understanding of the inter-relationships among PYD attributes, including the global PYD measure and individual dimensions, life satisfaction, and delinquency over time.

Regarding the first research question, results of correlation analyses and regression analyses consistently revealed that both the global PYD measure and individual PYD dimensions were negatively associated with adolescent delinquency, both concurrently and longitudinally. These findings initially supported our Hypothesis 1. The findings are congruent with the general theoretical expectation that the development of PYD attributes builds a constructive foundation for adolescent adaptive adjustment in the long run and protects them from externalizing life stress with delinquency (Shek et al., 2019a). Our findings also replicate previous empirical findings in the West (Geldhof et al., 2014; Voisin et al., 2020) and Chinese contexts (Shek and Lin, 2016; Shek and Zhu, 2018). As most existing empirical evidence in the Chinese context is from Hong Kong, the present findings further extend the conclusion to mainland China, suggesting that the general negative associations between PYD attributes and problematic development may hold across cultural contexts.

For the second research question, the overall findings also support our expectation that life satisfaction serves as a mediator for the predictions of PYD attributes on delinquency. In the regression models, each dimension of PYD attributes (i.e., “cognitive-behavioral competence,” “prosocial attribute,” “positive identity,” and “general PYD attribute”) and the total PYD score positively predicted life satisfaction, which subsequently functioned as a negative predictor of adolescent delinquency. These findings echo the previous evidence that different PYD attributes generally have positive linkages with life satisfaction (Lázaro-Visa et al., 2019; Ramos-Díaz et al., 2019) and life satisfaction negatively predict delinquency (Jung and Choi, 2017; Hanniball et al., 2018). In the separate regression analysis, all related PYD attributes also showed significant direct predictions on delinquency. This observation is in line with and extends the previous findings that involved Hong Kong Chinese adolescents and only investigated global measures of PYD (Sun and Shek, 2010, 2012).

With reference to the third research question, the separate regression analyses yielded consistent negative predictions of the global PYD measure and the four individual PYD dimensions. However, when all four individual dimensions of PYD attributes were included in a single model simultaneously, mixed findings were revealed. Specifically, positive identity and general PYD attribute showed expected negative predictions on delinquency via their positive effects on life satisfaction, whereas prosocial attributes showed insignificant to weak positive effects and cognitive-behavioral competence had even negative effects on life satisfaction. These findings support the conjecture of a nuanced relationship between individual dimensions of PYD and delinquency (Arbeit et al., 2014; Geldhof et al., 2014) and reinforce the importance of distinguishing between different PYD dimensions in examining their relationships with developmental outcomes. Several aspects of the findings are discussed below.

First, cognitive-behavioral competence, which mainly refers to adolescents’ intellectual and decision-making ability, showed negative cross-sectional and longitudinal predictions on life satisfaction. Although initially counterintuitive, this finding kindly mirrors the previous finding that caring, one C in Lerner’s (Lerner et al., 2011). Five Cs model (“connection,” “confidence,” “competence,” “character,” and “caring”), was positively related to anxiety and depressive symptoms when the effects of other Cs were statistically controlled (Holsen et al., 2017). It is argued that a high level of caring may represent adolescents’ emotional hypersensitivity that may make them manifest stronger anxiety and depressive feelings (Holsen et al., 2017).

Likewise, strong cognitive capacity may render adolescents more critical, more likely to experience over-expectations from others, and harder to feel satisfied with current life situations. Indeed, the setting of especially high standards (e.g., perfectionism) has been found to be associated with psychological distress and mental health problems (Proctor et al., 2009). Besides, higher cognitive-behavioral competence may also expose adolescents to the experimentation of risk behavior, which is believed by some scholars to be developmentally appropriate and adaptive (Dworkin, 2005; Shek and Lin, 2016). In essence, empirical findings support a certain degree of overlap between adaptive functioning and adolescent risk trajectories (Lewin-Bizan et al., 2010b; Warren et al., 2016). While original PYD theories hold that “one good thing leads to another” (Lewin-Bizan et al., 2010a, p. 759), these findings collectively suggest the need to refine the understanding of what is meant by “good” and optimal development.

Second, the prosocial attribute at Wave 1 showed a weak positive linkage with concurrent life satisfaction, and it did not have a significant association with life satisfaction over time. In contrast, positive identity and general PYD quality showed relatively stronger and robust associations with life satisfaction, both concurrently and longitudinally. In particular, the general PYD attribute was the strongest predictor that exerted the strongest indirect effect on delinquency through life satisfaction among all the PYD dimensions and showed the only significant direct effect on delinquency.

The findings do not imply that prosocial attribute (e.g., adopting of prosocial attitude and willingness to engage in prosocial behaviors) is not important, as it was a significant predictor of life satisfaction and delinquency in the current separate regression model and previous research (Schludermann et al., 2000; Lázaro-Visa et al., 2019). Instead, it can be reasoned that positive identity and general PYD attribute, especially the latter, may prevail over prosocial attributes in explaining the development of adolescent delinquency. Another explanation for the weak or insignificant effect of prosocial attributes on life satisfaction is that there is a “dark side” of prosociality, as being prosocial and helping others may lead to additional psychological costs and stress, which harms individual well-being (Bolino and Grant, 2016). These speculations should be verified in future studies.

For general PYD attribute, solid associations have been documented between those psychosocial competencies included in this dimension (e.g., bonding with parents, emotional skills, spirituality, resilience, and moral competence) and life satisfaction and delinquency in both Western and Chinese contexts (Raaijmakers et al., 2005; Shek and Zhu, 2018; Lázaro-Visa et al., 2019; Ramos-Díaz et al., 2019; Zhu and Shek, 2020b), which may contribute to the unique strong predictions of general PYD attribute in the present study. The strong and robust predictions of general PYD attribute also echo previous findings on the effect of Five Cs (“connection,” “confidence,” “competence,” “character,” and “caring”) (e.g., Geldhof et al., 2014), which are conceptually similar to those psychosocial competencies included in general PYD attribute. It is very likely that well-being, as indicated by high life satisfaction and low delinquency of mainland Chinese students, is also closely related to their competence in using adaptive intra- and inter-personal strategies such as the abilities to regulate emotions, overcome adversity, and build positive relationships with parents (Lázaro-Visa et al., 2019).

For positive identity, it showed relatively stable positive predictions on adolescent life satisfaction in the present study, although its effect was not as strong as that of the general PYD attribute. Another recent study involving mainland Chinese adolescents also found that positive self-identity was negatively associated with developmental problems (Xie et al., 2019; Chi et al., 2020). Although representation of the self has not been emphasized as much as interdependence in traditional Chinese culture (Greenfield et al., 2006; Yang and Zhou, 2017), a positive self-identity can be a protective factor of adolescent adjustment in mainland China in the contemporary society. Perhaps, the present Chinese adolescents are likely to be influenced by Western values and have become more individualistic as a result of the rapid westernization and modernization in mainland Chinese societies (Steele and Lynch, 2013; Cai et al., 2018). Future research will certainly benefit from the replication of the present findings.

To sum up, our findings explicate the complex associations between PYD dimensions and delinquency, which echo previous findings that some aspects of PYD attributes may not necessarily have positive predictions on adolescent developmental outcomes (Holsen et al., 2017; Lázaro-Visa et al., 2019). For example, while empathy, self-esteem, and emotional competence showed positive predictions on life satisfaction, cognitive competence was not a significant predictor when all these positive attributes were analyzed together (Lázaro-Visa et al., 2019). It is possible that while PYD attribute, in general, is associated with positive adolescent development (Lewin-Bizan et al., 2010a), certain dimensions, such as caring and cognitive competence, are compatible with risk behavior among adolescents (Arbeit et al., 2014; Warren et al., 2016). Taken together, these findings reinforce the need to use a discrete measure of PYD in future studies, as merely using a global measure of PYD is unable to reveal the complexity of the effect of different PYD attributes.

Practically, our findings highlight the notion that the cultivation of PYD attributes among adolescents can foster their life satisfaction and reduce delinquent behavior. This is particularly informative for educators, policymakers, researchers, and teachers, who are finding effective ways to deal with the worldwide trend of growing developmental problems among adolescents (World Health Organization, 2019). In the mainland China context, Bao (2018) asserted that rapid social change has made the youth’s life more stressful and leads to more juvenile delinquency. Chen and Cheung (2020) also suggested that the likelihood of committing delinquent acts is high among some Chinese adolescents as they face high levels of academic pressure and relational strains in terms of parental and teacher blame and punishment as a result of their unsatisfactory school achievement. Based on the present findings, building up inner strengths among Chinese adolescents is a promising strategy to promote their well-being and protect them from delinquency. Despite rich PYD programs and related rigorous evaluation findings in the West (Catalano et al., 2004; Lerner et al., 2011), the development, implementation and, evaluation of youth programs based on evidence-based approach are still at the infant stage in mainland China (Shek et al., 2019b; Zhu and Shek, 2020a). Although there are evaluation studies showing the positive impact of PYD programs in Hong Kong (e.g., Ma et al., 2019; Shek and Zhu, 2020), evaluation of PYD programs in mainland China is not systematic. Our findings provide further theoretical support for the utilization of PYD programs in mainland China.

This study has several limitations. First, we only collected two waves of data. Future studies will benefit from collecting more waves of data in a longer time span, which can help delineate a comprehensive picture of the inter-relationships among considered variables over time, particularly regarding the influence of the mediators. Second, the data were collected only from four secondary schools in four cities. Future studies need to replicate the present findings in other places in mainland China. Third, only self-reported questionnaires were used in the present study. Different informants such as parents and teachers can be involved in future studies to draw a richer picture for the related topic. Fourth, we only considered one mediator (i.e., life satisfaction). In the present study, the direct effect of the general PYD attribute on delinquency was much larger than the indirect effect accounted by life satisfaction, which suggests the existence of other mediators. There is a need to investigate other possible mediators, such as social support or school engagement in future studies. Finally, the effect sizes of the statistically significant results were small to moderate. One may raise a concern about the overpowering due to the large sample size in the present study. Of note, small effect size is not uncommon in social sciences research, and it is generally smaller in longitudinal research than in cross-sectional research (Ferguson, 2009). Obviously, there is a need to replicate the present findings. Besides, it is necessary to distinguish between statistical significance and practical significance. The present findings offer theoretical and practical implications in promoting adolescent life satisfaction by promoting their inner strengths.

## Conclusion

This study addressed several research gaps in the extant literature regarding the association between PYD attributes and delinquency among adolescents. Consistent with our hypotheses, separate dimensions of PYD attributes were negatively associated with adolescent delinquency through the mediating effect of life satisfaction, both concurrently and longitudinally. Nevertheless, when all the four dimensions of PYD attributes were included in one mediating effect model, cognitive-behavioral competence was negatively associated with life satisfaction, exerting a positive effect on delinquency. Future longitudinal studies with a longer time span should focus on replicating the present findings in other Chinese and non-Chinese communities and further explore the underlying mechanisms, including different mediators and moderators.

## Data Availability Statement

The raw data supporting the conclusions of this article will be made available by the authors, without undue reservation, to any qualified researcher.

## Ethics Statement

The studies involving human participants were reviewed and approved by “Human Subjects Ethics Subcommittee” at The Hong Kong Polytechnic University. Written informed consent to participate in this study was provided by the participants’ legal guardian/next of kin.

## Author Contributions

XZ contributed to the design of the project, data collection, data interpretation of the work, drafted the work, and revised it based on the critical comments provided by DS. DS conceived the project, obtained the funding, and edited the manuscript. Both authors contributed to the article and approved the submitted version.

## Conflict of Interest

The authors declare that the research was conducted in the absence of any commercial or financial relationships that could be construed as a potential conflict of interest.
